# The association between borderline personality disorder, childhood trauma, neuroticism, and self-rated or clinician-rated functional impairment in euthymic bipolar disorder-1 patients

**DOI:** 10.3389/fpsyt.2024.1444583

**Published:** 2024-10-10

**Authors:** Esat Fahri Aydın, Tuğba Koca Laçin

**Affiliations:** ^1^ Department of Psychiatry, Atatürk University Faculty of Medicine, Erzurum, Türkiye; ^2^ Department of Psychiatry, Ankara Etlik City Hospital, Ankara, Türkiye

**Keywords:** bipolar disorder, psychosocial functioning, adverse childhood experiences, neuroticism, borderline personality disorder

## Abstract

**Introduction:**

In this study, we mainly evaluated the associations of borderline personality disorder (BPD), neuroticism, and childhood trauma with the self-rated and clinician-rated overall functional impairment levels of adult euthymic patients with bipolar disorder-1 (BD-1). In addition, we compared patient and healthy control groups regarding the levels of of childhood trauma, neuroticism, BPD and functional impairment.

**Methods:**

In total, 90 euthymic BD-1 patients and 90 healthy controls were enrolled. The Childhood Trauma Questionnaire–Short Form, the neuroticism subscale of the Eysenck Personality Questionnaire Revised–Abbreviated Form, the Borderline Personality Questionnaire, the Functioning Assessment Short Test, and the Sheehan Disability Scale were administered to the participants.

**Results:**

The study revealed that the levels of BPD, neuroticism, emotional abuse, physical abuse, global childhood trauma, self-rated overall functional impairment, all the subdomains of self-rated functional impairment, clinician-rated overall functional impairment, and all the subdomains of clinician-rated functional impairment (except leisure time) were significantly higher in the patients than those in the healthy controls (p < 0.05). Clinician-rated functional impairment levels were significantly correlated with levels of BPD (r = 0.555, p<0.001), neuroticism (r = 0.429, p < 0.001), global childhood trauma (r = 0.391, p <0.001), and all subtypes of childhood trauma except sexual abuse. Self-rated functional impairment levels were significantly correlated with levels of neuroticism (r= 0.289, p = 0.006), physical neglect (r = 0.213, p = 0.044), and BPD (r = 0.557, p < 0.001). In the regression analyses, the self-rated overall functional impairment levels were only significantly associated with the BPD feature levels (β = 0.319, p < 0.001) and the clinician-rated overall functional impairment levels were only significantly associated with the BPD feature levels (β = 0.518, p < 0.001).

**Conclusion:**

The present study’s findings suggest that BPD features should be addressed in psychosocial interventions aimed at ameliorating functional impairment in patients with BD-1. Only BPD features were associated with self-rated and clinician-rated overall functional impairment levels in the regression analyses in the BD-1 patients. Performing self-rated and clinician-rated functional impairment assessments in the same clinical trial may give rise to relevant findings in the future.

## 1 Introduction

Bipolar disorder (BD), a prevalent condition and one of the major causes of disability worldwide, is highly correlated with decreased life expectancy (1, 2). Patients with BD experience significant levels of functional impairment, even in remission. In a systematic review and meta-analysis of euthymic patients with BD, their overall functional impairment was 58.6% (3). A study conducted in seven countries using a large sample (n = 5,882) reported that functional impairment in patients with BD was between 41% and 75%. In this study, a higher number of mood episodes, decreased education levels, comorbid substance use disorder, and an increased number of psychotropic medications were found to be positively related to increased functional impairment (4). Considering the functional difficulties of patients with BD, identifying the predictors of functional impairment in these patients constitutes an important area of research.

Childhood trauma is a complex experience, and there is a growing interest in research about the consequences of childhood trauma (de Azambuja Farias et al., 2019). BD is associated with adverse childhood experiences, and one study showed that childhood trauma was 2.63 times more likely to occur in BD patients compared with non-clinical controls (5). BD patients with CT have a significantly higher risk of substance misuse disorder, an earlier age of bipolar onset, and a greater number of mood episodes compared to BD patients without CT (Agnew-Blais & Danese, 2016).

Neuroticism is considered one of the most significant personality features of BD. It is described as the tendency to experience negative emotions, such as sorrow, anxiety, irritability, guilt, loneliness, disappointment, and aggression, when faced with stressful events (6, 7). Neuroticism has been correlated with low well-being and depression (8, 9). It has been found to be genetically positively correlated with BD, anxiety disorders, major depressive disorder, insomnia, attention-deficit/hyperactivity disorder, and loneliness (7). In a previous study, patients with BD were associated with higher levels of neuroticism than healthy controls (10).

The main features of BPD are identity problems, difficulties in emotion regulation, and intense interpersonal problems that cause suffering (11). Patients with BPD suffer greatly from psychosocial impairment and its consequences (12, 13). BPD comorbidity in BD is a challenging issue in clinical settings, and a systematic review and meta-analysis found BPD comorbidity in BD to be 21.6% (14). BPD comorbidity is related to worse clinical features in BD (15). In a study with a large sample size (n = 375), self-reported BPD features were associated with more frequent episodes in BD (16).

Approximately 80% of BD patients do not have a BPD diagnosis (17). Therefore, an exploration of the effects of BPD features on the psychosocial functioning of patients with BD may reveal valuable information about specific BPD features. For example, in clinical assessments of personality disorders, BPD and neuroticism have shown the strongest associations (18), including that neuroticism and BPD share a common genetic background (19). In addition, individuals with BPD have been associated with greater childhood trauma than those with other personality disorders (20). Moreover, a meta-analysis revealed that individuals with BPD have an elevated experience of childhood trauma compared to healthy controls (13.91 times more) and those with other psychiatric disorders (3.15 times more) (21). Regarding the sub-types of childhood trauma, emotional abuse and neglect were higher in BPD compared to healthy control participants (21). Additionally, neuroticism has been associated with childhood traumatic events (22, 23). These results indicate that associations exist between BPD, childhood traumatic events, and neuroticism. Regarding BD, as previously mentioned, BPD comorbidity in BD is at a non-negligible level, and the levels of neuroticism and childhood trauma are higher in BD than those in healthy controls.

The above-mentioned associations between childhood trauma, BPD, and neuroticism and their relation to BD suggest the need to explore the effects of these variables on functional impairment levels in BD. Therefore, in the present study, we aimed to explore whether the features of BPD, neuroticism, and childhood trauma predict functional impairment levels in patients with BD-1. To our knowledge, to date, the predictive effects of childhood trauma, BPD, and neuroticism on the functional impairment levels of a sample of pure euthymic adult bipolar disorder-1 (BD-1) patients have not been explored. In addition, the present study is the first to perform functional impairment evaluation of BD using self-reported and clinician-reported instruments. Additionally, we aimed to compare patients with BD-1 and healthy controls regarding childhood trauma, BPD features, neuroticism, and functional impairment. The present study’s main hypotheses are as follows: 1) The BD-1 group will have higher levels of neuroticism, functional impairment, global childhood trauma, sub-types of childhood trauma, and features of BPD, and 2) High levels of neuroticism, global childhood trauma, and sub-types of childhood trauma, and features of BPD will be associated with high levels of overall functional impairment of patients with BD-1.

## 2 Materials and methods

Ninety euthymic patients with BD-1 and 90 healthy controls were enrolled in our outpatient clinic between February 2020 and May 2022. The study’s second author assessed all the participants. In line with DSM-IV-TR criteria, the patient group’s diagnoses were verified by the Turkish version of the SCID-I (24, 25). Ethical approval for the current study was granted by the Atatürk University Clinical Research Ethical Committee (Date: 16/01/2020, meeting number: 01, and decision no: 03). All the participants’ informed consent was obtained before the study commenced.

The inclusion criteria were as follows: BD-1 diagnosis with at least eight weeks of remission, between the ages of 18 and 65, and enough intellectual capacity to read and assess the self-report scales. Remission was defined as a score of ≤ 7 on the Turkish version of the Hamilton Depression Rating Scale 17-item (HAM-D-17) (26, 27) and a score of ≤ 5 on the Turkish version of the Young Mania Rating Scale (YMRS) (28, 29). The exclusion criteria for patients with BD-1 were intellectual disability, current pregnancy or lactation, any comorbid psychiatric diagnosis, according to the DSM-IV, within the previous 12 months, and any medical illness affecting their general medical status. The healthy controls were drawn from hospital staff and their relatives with no history of mental disorder and no medical status affecting their general medical condition.

A sociodemographic clinical data form developed by the present study’s researchers was used to obtain the necessary data from the participants in relation to the study’s aims. BPD features were assessed by the Borderline Personality Questionnaire, childhood traumatic events were assessed by the Childhood Trauma Questionnaire–Short Form, and neuroticism was assessed by neuroticism subscale of The Eysenck Personality Questionnaire Revised–Abbreviated Form. The participants’ functional impairment levels were evaluated using the Sheehan Disability Scale and the Functioning Assessment Short Test.

The Childhood Trauma Questionnaire–Short Form (CTQ-28) is a self-report questionnaire developed by Bernstein et al. (30) that measures childhood trauma across 28 items. The scale includes five subscales: emotional abuse, emotional neglect, physical abuse, physical neglect, and sexual abuse. The total scores on the scale imply global childhood trauma. Higher total scores mean elevated levels of global childhood trauma, and higher subscale scores represent higher subscale features. The Turkish reliability and validity tests of the scale were performed by Şar et al. (31).

The Eysenck Personality Questionnaire Revised–Abbreviated Form (EPQR-AF) is a self-report questionnaire that evaluates three personality features: neuroticism, psychoticism, and extroversion (32). In the present study, only the neuroticism subscale (EPQR-AF-neuroticism) was administered to the participants. Higher scores mean elevated levels of neuroticism. The Turkish reliability and validity tests of the scale were performed by Karancı et al. (33).

The Borderline Personality Questionnaire (BPQ) is a self-rated instrument comprising 80 items developed by Poreh et al. (34). The total score of the BPQ is the sum of all its items. Higher total scores on the BPQ represent higher BPD features. The Turkish reliability and validity tests of the BPQ were performed (35).

The Sheehan Disability Scale (SDS) is a self-rated scale with three questions and three dimensions (36): functioning of family life, social life, and work. The SDS’s total score is the sum of the scores of its three questions and relates to overall functional impairment. Higher total scores represent higher overall functional impairment, and higher subscale scores represent higher functional impairment levels in the subscales (37, 38).

The Functioning Assessment Short Test (FAST) is a clinician-rated instrument developed by Rosa et al. (39). The FAST includes 24 items and assesses psychosocial functioning in six domains: leisure time, interpersonal relationships, financial issues, cognitive functioning, occupational functioning, and autonomy. The sum of all the items indicates overall functional impairment. Higher total scores imply more severe overall functional impairment, and higher subscale scores imply higher functional impairment levels of the subscale domains. The Turkish reliability and validity tests of the FAST were conducted (40).

The study data underwent statistical analysis using the Statistical Package for Social Sciences (SPSS) for the Windows 25 package program. Normalization of the distribution of numerical data was analyzed using the Shapiro-Wilk and Kolmogorov-Smirnov tests. General descriptive statistics, such as median, interquartile range values of continuous variables and frequency, and percentage values of categorical variables were obtained. Discrete distribution analysis between the groups was performed using the chi-square test. For continuous variables in the analysis of differences between the groups, the Mann-Whitney U test was used for non-normally distributed data. Spearman’s Rho correlation tests were used for the correlation analysis of non-normally distributed and ordinal data. Linear regression analysis was performed using the stepwise selection method to determine the independently associated variables with FAST total and SDS total variables in the BD-1 group. The confidence interval of the results was evaluated as 95%. In all the analyses, the results were considered significant at p < 0.05.

## 3 Results

### 3.1 Clinical and demographic features

In total, 90 patients with BD-1 and 90 healthy controls were enrolled in the present study. The median age of the patients was 35 (19.3) years, and the median years of education was 12 (6.3) years. Of the patients, 51.10% (n = 46) were female. The patient group (n = 90) and healthy control group (n = 90) were statistically similar in terms of age, years of education, and gender (p > 0.05). The positive family history of BD was 33.30% (n = 30) in the patient group, whereas the positive family history of BD was 1.1% (n = 1) in the healthy control group. The patient group had a significantly higher family history of BD than the control group (χ 2 = 30.552, p < 0.001). The positive history of episodes with psychotic features was 70.00% (n = 63), and the positive history of suicide attempts was 22.22% (n = 20) in the patient group. In the comparison of the two groups regarding subclinical symptoms, the BD-1 group showed significantly higher HAM-D-17 (p < 0,001) and YMRS (p < 0.001) scores. The sociodemographic and clinical variables of the participants are summarized in **Table 1**.

**Table 1:** Sociodemoghraphic and clinical variables of participants.

Descriptive characteristics	BD-1 group (n=90)	Control group (n=90)	Statistical test
**Age, *Median (IQR)***	35 (19.3)	35.5 (24.3)	U=4115.5 p=0.851
**Education level (years), *Median (IQR)***	12 (6.3)	12 (7.3)	U=4279.5 p=0.507
**Male, *n(%)***	44 (48.90)	41 (45.60)	χ 2 = 0.201 p=0.654
**Female, *n(%)***	46 (51.10)	49 (54.40)	
**Single, *n(%)***	39 (43.30)	31(34.40)	χ 2 = 7.642 **p=0.022**
**Married, *n(%)***	42 (46.70)	57(63.30)	
**Widow/Divorced, *n(%)***	9 (10.00)	2 (2.20)	
**Employed, *n(%)***	35 (38.90)	54 (60.00)	χ 2 = 19.236 **p<0.001**
**Unemployed, *n(%)***	40 (44.40)	18 (20.00)	
**Retired, *n(%)***	10 (11.10)	4 (4.40)	
**Student, *n(%)***	5 (5.60)	14 (15.60)	
**Positive family history of psychiatric disorder, *n (%)***	46 (51.1)	12 (13.3)	χ 2 = 27.702 **p<0.001**
**Positive family history of BD, *n(%)***	30 (33.30)	1 (1.10)	χ 2 = 30.552 **p<0.001**
**Age of onset of disorder, *Median (IQR)***	21.5 (9.3)	–	
**Duration of disorder (months), *Median (IQR)***	126 (198)	–	
**Number of hospitalizations, *Median (IQR)***	2 (2)	–	
**Duration of remission (months), *Median (IQR)***	16.5 (43.3)	–	
**Total number of episodes, *Median (IQR)***	4 (4)	–	
**Positive history of episode with** **psychotic features, *n (%)***	63 (70.00)	–	
**Positive history of suicide attempts, *n (%)***	20 (22.22)	–	
**HAM-D-17 Score, *Median (IQR)***	2 (3.0)	0 (1.0)	U=2387.5 **p<0.001**
**YMRS Score, *Median (IQR)***	1 (3.0)	0 (0.0)	U=2321.5 **p<0.001**

Chi-Squared Test and Mann-Whitney’s U Test. U: Mann-Whitney’s U Test; χ 2: Chi-Square Test. Significant outcomes of p-values are reported in bold. Bold values represent p<0.05. HAM-D-17, Hamilton Depression Rating Scale 17-item version; IQR, Interquartile Range; YMRS, Young Mania Rating Scale.

### 3.2 Hypothesis 1: The BD-1 group will have higher levels of neuroticism, functional impairment, global childhood trauma, sub-types of childhood trauma, and features of BPD than the healthy controls

The emotional abuse (p < 0.001) and physical abuse (p = 0.031) subscales of CTQ-28 and CTQ-28 total (p = 0.008) scores were significantly higher in the BD-1 group than in the healthy control group. Regarding the features of neuroticism, the EPQR-AF scores of the BD-1 group were significantly higher than those of the healthy control group (p < 0.001). Additionally, the BPQ total (p = 0.015) scores of the patients with BD-1 were significantly higher than the scores of the healthy controls.

Regarding self-rated functional impairment, in the SDS total scores (p < 0.001) and all domains of the SDS scores (i.e., family life, social life, and work) (p < 0.001), the BD-1 group showed significantly higher scores than the healthy controls. On the basis of interviewer-rated functional impairment, except for the leisure time subscale, in all the subscales of the FAST (i.e., interpersonal relationships, financial issues, cognitive functioning, occupational functioning, and autonomy) (p < 0.001) and the FAST total scores (p < 0.001), those of the BD-1 group were significantly higher than in the healthy control group. The results of the comparison between the two groups are presented in **Table 2**.

**Table 2:** Comparison of scores of BPQ, CTQ-28, EPQR-AF, FAST, and SDS between the groups.

	BD-1 group (n=90)	Control group (n=90)	Statistical test
	Median (IQR)	Median (IQR)	
**CTQ-28 Emotional Abuse**	6.0 (3.0)	5.0 (1.0)	U=2906.5 **p<0.001**
**CTQ-28 Physical Abuse**	5.0 (1.0)	5.0 (0.0)	U=3485.5 **p=0.031**
**CTQ-28 Sexual Abuse**	5.0 (0.0)	5.0 (0.0)	U=3731.5 p=0.072
**CTQ-28 Emotional Neglect**	9.0 (7.0)	6.0 (4.0)	U=3593 p=0.189
**CTQ-28 Physical Neglect**	7.0 (4.0)	8.5 (5.0)	U=3554 p=0.146
**CTQ-28 Total**	34.5 (14.0)	31.0 (10.0)	U=3130.5 **p=0.008**
**EPQR-AF Neuroticism**	2.0 (2.0)	0.5 (3.0)	U=2812.5 **p<0.001**
**BPQ Total**	22.5 (18.0)	17.0 (10.0)	U=3204** p=0.015**
**SDS Family life**	3.0 (5.0)	0.0 (2.0)	U=2095** p<0.001**
**SDS Social life**	4.0 (3.5)	0.0 (2.0)	U=2018** p<0.001**
**SDS Work**	5.0 (6.0)	0.0 (1.0)	U=1658** p<0.001**
**SDS Total**	11.0 (12.3)	0.0 (5.0)	U=1482.5 **p<0.001**
**FAST-Leisure**	2.0 (2.0)	1.0 (2.0)	U=3476.5 p=0.088
**FAST-Interpersonal**	2.0 (6.0)	0.0 (2.0)	U=2561.5 **p<0.001**
**FAST-Financial**	0.0 (2.0)	0.0 (0.0)	U=2773.5 **p<0.001**
**FAST-Cognitive**	3.0 (5.3)	0.0 (2.3)	U=2277** p<0.001**
**FAST-Occupational**	1.0 (6.0)	0.0 (1.0)	U=2908** p<0.001**
**FAST-Autonomy**	2.0 (3.0)	0.0 (1.0)	U=2661.5 **p<0.001**
**FAST-Total**	12.0 (17.5)	3.0 (9.0)	U=2035** p<0.001**

Mann-Whitney’s U Test. U: Mann-Whitney’s U Test. Significant outcomes of p-values are reported in bold. Bold values represent p<0.05. BPQ, Borderline Personality Questionnaire; CTQ-28, Childhoood Trauma Questionnaire Short Form; EPQR-AF, The Eysenck Personality Questionnaire Revised-Abbreviated Form; FAST, Functioning Assessment Short Test; IQR, İnterquartile Range; SDS, Sheehan Disability Scale.

### 3.3 Hypothesis 2: High levels of neuroticism, global childhood trauma, sub-types of childhood trauma, and features of BPD will be associated with high levels of functional impairment in patients with BD-1

The FAST total scores were significantly correlated with the scores of EPQR-AF- neuroticism (r = 0.429, p < 0.001), the CTQ-28 total (r = 0.391, p < 0.001), and except for the scores of CTQ-28 sexual abuse, all the subscale scores of the CTQ-28 and the scores of BPQ total (r = 0.555, p < 0.001). The SDS total scores were significantly correlated with the scores of EPQR-AF-neuroticism (r = 0.289, p = 0.006), CTQ-28 physical neglect (r = 0.213, p = 0.044), and BPQ total (r = 0.557, p < 0.001). Regarding the association between childhood trauma and personality features, the EPQR-AF-neuroticism scores of the patients were significantly correlated with the scores of the CTQ-28 total (r = 0.227, p = 0.031), CTQ-28 emotional abuse (r = 0.315, p = 0.002), and CTQ-28 sexual abuse (r = 0.215, p = 0.042). Additionally, the BPQ total scores were significantly correlated with the scores of the CTQ-28 total (r = 0.409, p < 0.001), CTQ-28 emotional abuse (r = 0.363, p < 0.001), CTQ-28 physical neglect (r = 0.389, p < 0.001), and CTQ-28 emotional neglect (r = 0.283, p = 0.007). The BPQ total scores were significantly correlated with the scores of EPQR-AF-neuroticism (r = 0.554, p < 0.001). HAM-D-17 scores were significantly correlated with the scores of CTQ-28 Total (r = -0.222, p = 0.036) and CTQ-28 physical neglect (r = -0.236, p = 0.025). YMRS scores were significantly correlated with the scores of FAST total (r = -0.213, p = 0.044), EPQR-AF-neuroticism (r = -0.230, p = 0.030) and CTQ-28 physical neglect (r = -0.212, p = 0.045). The results of these correlations are presented in **Table 3**.

**Table 3:** Spearman’s Rho correlation analysis of FAST, SDS, CTQ-28, BPQ, EPQR-AF-neuroticism, HAM-D-17, YMRS scores in the BD-1 group (n=90).

		FAST Total	SDS Total	CTQ-28 Total	BPQ Total	EPQR-AF Neuroticism	HAM-D-17	YMRS
**EPQR-AF Neuroticism**	**r**	0.429**	0.289**	0.227*	0.554**	–	0.028	-0.230*
	**p**	**<0.001**	**0.006**	**0.031**	**<0.001**	**-**	0.790	**0.030**
**CTQ-28 Total**	**r**	0.391**	0.203	–	0.409	0.227*	-0.222*	-0.133
	**p**	**<0.001**	0.055	–	**<0.001**	**0.031**	**0.036**	0.210
**CTQ-28 Emotional abuse**	**r** **p**	0.320** **0.002**	0.140 0.189	0.779** **<0.001**	0.363 **<0.001**	0.315** **0.002**	-0.186 0.079	-0.195 0.066
**CTQ-28 Physical abuse**	**r** **p**	0.264* **0.012**	0.094 0.378	0.597** **<0.001**	0.116 0.275	0.099 0.351	-0.035 0.743	-0.002 0.988
**CTQ-28 Sexual abuse**	**r** **p**	0.202 0.056	0.113 0.287	0.326** **0.002**	0.159 0.134	0.215* **0.042**	0.052 0.623	-0.102 0.337
**CTQ-28 Physical neglect**	**r** **p**	0.372** **<0.001**	0.213* **0.044**	0.677** **<0.001**	0.389** **<0.001**	0.123 0.248	-0.236* **0.025**	-0.212* **0.045**
**CTQ-28 Emotional neglect**	**r** **p**	0.269* **0.010**	0.106 0.321	0.836** **<0.001**	0.283** **0.007**	0.129 0.226	-0.190 0.073	-0.106 0.321
**BPQ Total**	**r** **p**	0.555** **<0.001**	0.557** **<0.001**	0.409** **<0.001**	- -	0.554** **<0.001**	-0.091 0.395	-0.140 0.189
**HAM-D-17**	**r** **p**	-0.045 0.675	0.068 0.526	-0.222* **0.036**	-0.091 0.395	0.028 0.790	- -	0.154 0.148
**YMRS**	**r**	-0.213*	-0.200	-0.133	-0.140	-0.230*	0.154	–
	**p**	**0.044**	0.059	0.210	0.189	**0.030**	0.148	**-**

r: Spearman’s rho correlation coefficient. Significant outcomes of p-values are reported in bold. Bold values represent p<0.05. * p<0.05, ** p <0.01. BPQ, Borderline Personality Questionnaire; CTQ-28, Childhood Trauma Questionnaire Short Form; EPQR-AF, The Eysenck Personality Questionnaire Revised-Abbreviated Form; FAST, Functioning Assessment Short Test; HAM-D-17, Hamilton Depression Rating Scale 17-item version; SDS, Sheehan Disability Scale; YMRS, Young Mania Rating Scale.

In the present study, we aimed to identify the factors associated with clinician-rated and self-rated functional impairment of patients with BD-1. We performed two regression analyses to assess the predictors of overall functional impairment levels in patients with BD-1. In one analysis, the dependent variable was the SDS total; in the other, the dependent variable was the FAST total. The variables that were significantly correlated with SDS total and FAST total were evaluated in the regression analyses. HAM-D-17 and YMRS scores included as covariates in the regression analyses.

Only the SDS total scores were significantly associated with the BPQ total scores (β = 0.319, p < 0.001). Other scores included in the analysis, EPQR-AF-neuroticism (p = 0.663), CTQ-28 physical neglect (p = 0.829), HAM-D-17 (p = 0.213), and YMRS (p = 0.059) were found to be non-significant.

Only the FAST total scores were significantly associated with the scores of the BPQ total (β = 0.518, p < 0.001). Other scores included in the analysis, EPQR-AF neuroticism (p = 0.115), CTQ-28 total (p = 0.071), CTQ-28 emotional abuse (p = 0.867), CTQ-28 physical abuse (p = 0.063), CTQ-28 physical neglect (p = 0.121), CTQ-28 emotional neglect (p = 0.956), HAM-D-17 (p = 0.705), and YMRS (p = 0.602) were found to be non-significant. The results of the regression analyses of the above parameters are presented in **Tables 4**, **5**.

**Table 4:** Independently associated variables with SDS-total in linear regression analysis performed in the BD-1 group.

		β	SE	t	p-value	Lower limit	Upper limit
Constant		3.782	1.360	2.781	0.007	1.080	6.484
BPQ Total		0.319	0.050	6.326	**<0.001**	0.219	0.419

Dependent variable: SDS- total. Adjusted R^2^ = 0.305, F=40.020, p<0.001. Bold values represent p<0.05. SDS, Sheehan Disability Scale; BPQ, Borderline Personality Questionnaire.

**Table 5:** Independently asssociated variables with FAST-total in linear regression analysis performed in the BD-1 group.

		β	SE	t	p-value	Lower limit	Upper limit
Constant		3.056	2.347	1.302	0.196	-1.609	7.721
BPQ Total		0.518	0.087	5.955	**<0.001**	0.345	0.692

Dependent variable: FAST total. R^2^=0.279, F=35.461, p<0.001. Bold values represent p<0.05. FAST, Functioning Assessment Short Test; BPQ, Borderline Personality Questionnaire.

## 4 Discussion

In the present study, only BPD features were associated with the overall functional impairment levels of patients with BD-1 through clinician-rated and self-rated assessments. The BD-1 group showed higher levels of emotional abuse, physical abuse, global childhood trauma, neuroticism, and features of BPD. Regarding both the self-rated and clinician-rated assessments of psychosocial functioning in the present study, the levels of overall functional impairment of the patients were higher than those of the healthy controls. Additionally, in the evaluations of all subdomains of the self-rated assessments and clinician-rated assessments (except leisure time), patients with BD-1 had higher levels of functional impairment than the healthy controls.

In a recent study of 345 fully or partially remitted patients with BD, the patient group had significantly higher levels of global childhood trauma and all sub-types of childhood trauma (i.e., emotional abuse, emotional neglect, physical abuse, physical neglect, and sexual abuse) than the healthy controls (41). As previously mentioned, a meta-analysis revealed the importance of childhood trauma in BD (5). In the present study, global childhood trauma and the emotional and physical abuse levels of the patients were significantly higher than those of the healthy controls. In addition, the neuroticism levels of the patients in the present study were significantly higher than those of the healthy controls. Similarly, a recent study from Denmark showed that the neuroticism levels of patients with BD were considerably higher than those of the healthy controls (42). In the present study, we found that the BPD features of the patient group were significantly higher than those of the healthy controls. A recent nationwide study showed that in inpatients with BD, the comorbid personality disorder rate was 12.2%, with BPD showing the highest rate among these disorders, at 8.2% (43). All the above-mentioned results regarding childhood trauma, neuroticism, and BPD features imply that these factors should be considered in the routine practice of clinicians dealing with patients with BD-1.

In the present study, we assessed the participants’ psychosocial functioning using a self-rated scale (SDS) and a clinician-rated scale (FAST). In both the self-rated and clinician-rated assessments, BD-1 patients’ overall functional impairment levels were significantly higher than those of the healthy controls. Additionally, except for the leisure time subscale of the FAST, all the subdomains of the self-rated and the clinician-rated assessments of patients with BD-1 showed significantly lower functioning levels than those of the healthy controls. A recent study from Ethiopia using the same clinician-rated assessment tool (FAST) that the present study used to assess psychosocial functioning reported that leisure time was the least impaired functional domain in patients with BD (44). However, in that study, a comparison of healthy controls and BD patients was not performed. A previous study reported that BD patients had higher functional impairment levels than healthy controls (41). However, to our knowledge, the present study is the first to compare the psychosocial functioning of BD-1 patients using both clinician-rated and self-rated assessment scales. The results of both these assessments reveal significant functional impairment levels in patients with BD-1.

Previous studies have shown a positive association between increased childhood trauma and decreased psychosocial functioning in patients with BD (45, 46). Additionally, in a recent study, increased childhood trauma levels were independently associated with levels of increased functional impairment in partially or fully remitted patients with BD compared to healthy controls. In that study, the levels of physical and emotional neglect were positively associated with the functional impairment levels (41). In the present study, global childhood trauma levels were correlated with only clinician-rated functional impairment levels. Regarding the sub-types of childhood trauma, emotional abuse, physical abuse, physical neglect, and emotional neglect were correlated with clinician-rated functional impairment levels. Self-rated functional impairment levels were correlated with only physical neglect levels. However, in the regression analyses of the present study, no independent association was found between functional impairment levels and the global or sub-types of childhood trauma. Although the present study did not reveal significant results in relation to childhood trauma and functional impairment, the above-mentioned previous studies (41, 45, 46) revealed significant outcomes between childhood trauma and functional impairment in BD patients, which should be taken into account in future clinical trials.

In the present study, in the clinician-rated assessments and self-rated assessments, neuroticism was correlated with overall functional impairment in patients with pure BD-1. However, in the regression analyses, neuroticism was not associated with self-rated and clinician-rated functional impairment levels. A previous study showed an association between neuroticism and psychosocial difficulties in BD (47). However, in the mentioned study (47), the participants had other conditions besides BD-1. Neuroticism is characterized by a tendency to experience undesirable emotions, difficulty controlling emotions, and dysfunctional behavior and cognition under stress (48). Thus, patients with high neuroticism levels are likely to experience problematic symptoms in stressful situations. Difficulty coping in these challenging situations may result in their functional impairment. Therefore, it is crucial to evaluate the association between neuroticism and functional impairment in BD-1 in future clinical trials.

Behavioral control problems and emotion regulation difficulties are characteristic patterns of BPD. Impulsive behaviors mostly appear in stressful conditions, and these behaviors function as a way of managing emotional instability (49). In addition, evaluating others’ trustworthiness may constitute a problem in BPD, particularly in social interactions characterized by high stress levels (50). Additionally, in stressful conditions, individuals with BPD can quickly generate paranoid ideas, often experiencing sudden changes in the way they view themselves and other people, such as all-good or all-bad (51). Patients with BPD tend to use immature defense mechanisms (e.g., projection, projective identification, acting out, and splitting) (52). Defense mechanisms are psychologically automatic behaviors that are used to overcome anxiety, and the use of immature defense mechanisms is related to problems of adaptation and psychosocial functioning (53). BPD is mainly considered to be associated with insecure attachment features (54, 55), and secure attachment features negatively predict clinician-rated functional impairment levels in euthymic BD patients BD (56). Additionally, from a cognitive theory perspective, BPD’s main cognitive schemata are associated with the unacceptability and powerlessness of the self and the dangerousness of the world (57). As mentioned above, the dysfunctional features of BPD may lead to interpersonal difficulties and social adaptation difficulties. Due to these difficulties, BPD features may give rise to psychosocial functioning problems. In line with this, in the exploration of the National Epidemiologic Survey on Alcohol and Related Conditions (NESARC) data with 34,481 participants, a BPD diagnosis was associated with functional impairment (58).

Comorbidity of BPD with BD is a widespread occurrence, and nearly one-fifth of patients with BD exhibit BPD comorbidity (14, 59). Data from waves 1 and 2 of the longitudinal National Epidemiologic Survey on Alcohol and Related Conditions (NESARC) revealed that BPD comorbidity in BD-1 was 29.0% and was associated with higher levels of childhood adversities and worse clinical outcomes than BD without BPD comorbidity (60). The 2012–13 National Epidemiological Survey on Alcohol and Related Conditions (NESARC-III) data exploration revealed that BD patients with BPD comorbidity were associated with more disadvantageous results than BD patients without BPD comorbidity regarding social life, economic conditions, and physical health (61). Additionally, in a systematic review, BPD’s impairment on individuals’ professional functioning was found to be comparable with BD (62). Previously, in a study of adolescents with BD, higher BPD features were associated with higher functional impairment (63). However, in this clinical trial, the participants were not only adult BD-1 patients. In the present study, increased BPD feature levels predicted self-rated and clinician-rated functional impairment levels in adult euthymic patients using a sample of patients with pure BD-1. Moreover, in the present study, self-reported BPD features were the only predictor of functional impairment levels in self-rated or clinician-rated assessments.

Currently, symptomatic improvement goals are insufficient for patients and clinicians in the follow-up of BD patients. In line with clinicians’ efforts, patients with BD wish to live their daily lives more satisfactorily. Thus, there is a vast effort to define and measure psychosocial functioning more correctly and efficiently in BD. As a result of the studies on this subject, the assessment of psychosocial functioning in patients with BD was suggested to be performed in three different evaluation patterns. First, from the perspective of patients (self-rated), second, from the perspective of clinicians (clinician-rated), and third, using a performance-based objective tool (64). In previous studies, only clinician-rated *or* self-rated assessment tools have been used. A strength of the present study is its use of both self-rated (SDS) and clinician-rated (FAST) tools to assess psychosocial functioning. However, the lack of an objective tool used to determine functional impairment is an aspect of the present study that needs improvement. On the other hand, the two psychosocial functioning assessment instruments—SDS (65) and FAST (39)—that we used in the present study have been previously validated for BD. The inclusion of a healthy control group ensured that the BD-1 group could be compared according to the study’s aims. Additionally, to our knowledge, this is the first study to explore the associations of functional impairment with BPD, neuroticism, and childhood trauma in a sample of only euthymic adult patients with BD-1.

The present study has the following limitations. First, as previously mentioned, the study lacks a performance-based functional impairment assessment. Second, the features of neuroticism, BPD, and childhood trauma were only assessed by self-rated instruments. In the future, these variables could also be evaluated by clinical interviews and clinician-rated instruments. Third, the cross-sectional design of the present study did not allow us to infer conclusions regarding the associations that the present study explored. Fourth, the association between psychosocial functioning and BPD may be deemed circular; that is, with the adverse influences of functional impairment, patients’ features of BPD may be aggravated. Future studies could be better designed to reveal causality directions between psychosocial functioning and the associated variables in BD that were explored in the present study. Thus, longitudinal follow-up studies are necessary to explore these causal effects in patients with BD. Fifth, recall bias may have caused a hesitation in the truthfulness of the retrospective assessments of childhood traumatic events. To explore the recall bias effect, a previous study was performed with patients with BD. In an interval of 18 months, the CTQ-28 was administered to the participants, and reasonable test-retest reliability was found (66). Sixth, the brain’s different regions may be affected differently at different ages (67), and the present study did not assess the specific periods when the traumatic experiences occurred. Seventh, a recent meta-analysis showed that the features of personality disorders may decline over time, and BPD is not as stable a personality disorder as, for example, the obsessive-compulsive, schizoid, and antisocial personality disorders (68). Over a long period, different assessments might ensure more objective results regarding feature assessments of personality disorders than were attained in the present study. Eighth, enrollment of the participants only in the outpatient clinic at a single site may impede the generalizability of the results. Ninth, the exclusion of non-euthymic patients with BD may affect the generalizability of the present study’s results. Tenth, comorbid psychiatric disorders are not rare in patients with BD; therefore, excluding comorbid psychiatric disorders may also affect the generalizability of the present study’s results. Eleventh, not including BD type-2 may also impede the generalizability of the present study’s results regarding BD. Finally, even though the patients were in remission, all the patients received psychotropics. The possible influences of the medications on the patients’ functional impairment levels were not evaluated. Evaluation of the medications’ side effects on patients’ functional impairment levels could have helped obtain more detailed results.

## 5 Conclusion

In summary, the present study showed that BPD features might have a role in functional impairment in euthymic patients with BD-1. Only BPD features were independently associated with functional impairment levels using clinician-rated and self-rated assessment tools in the regression analyses. Regarding the results of the present study, the features of BPD may need to be evaluated in the follow-up of patients with BD-1. Mentalization-based therapy and dialectical behavior therapy effectively treat BPD features (69, 70). Therefore, the adverse effects of BPD on the psychosocial functioning of euthymic patients with BD-1 may be tackled using the above-mentioned psychotherapy methods. Considering the high levels of psychosocial functioning problems in patients with BD-1, even in remission, these findings may assist clinicians in intervening in the functional impairment of patients with BD-1.

## Data Availability

The raw data supporting the conclusions of this article will be made available by the authors, without undue reservation.
